# Rare Missense Variants of the Human β4 Subunit Alter Nicotinic α3β4 Receptor Plasma Membrane Localisation

**DOI:** 10.3390/molecules28031247

**Published:** 2023-01-27

**Authors:** Sara Francesca Colombo, Cecilia Galli, Arianna Crespi, Massimiliano Renzi, Cecilia Gotti

**Affiliations:** 1 CNR Institute of Neuroscience, 20854 Vedano al Lambro, Italy; 2 NeuroMi Milan Center for Neuroscience, University of Milano—Bicocca, 20126 Milan, Italy; 3 Department of Physiology and Pharmacology, “Sapienza" University of Rome, 00185 Rome, Italy

**Keywords:** α3β4 nicotinic subtype, nAChRs, stoichiometry, SNPs, α3β4 trafficking

## Abstract

α3β4 nicotinic acetylcholine receptors (nARs) are pentameric ligand-gated cation channels that function in peripheral tissue and in the peripheral and central nervous systems, where they are critical mediators of ganglionic synaptic transmission and modulators of reward-related behaviours. In the pentamer, two α3β4 subunit couples provide ligand-binding sites, and the fifth single (accessory) subunit (α3 or β4) regulates receptor trafficking from the endoplasmic reticulum to the cell surface. A number of rare missense variants of the human β4 subunit have recently been linked to nicotine dependence and/or sporadic amyotrophic lateral sclerosis, and altered responses to nicotine have been reported for these variants; however, it is unknown whether the effects of mutations depend on the subunit within the ligand-binding couples and/or on the fifth subunit. Here, by expressing single populations of pentameric receptors with fixed stoichiometry in cultured cells, we investigated the effect of β4 variants in the fifth position on the assembly and surface exposure of α3β4 nAChRs. The results demonstrate that the missense mutations in the accessory subunit alone, despite not affecting the assembly of α3β4 receptors, alter their trafficking and surface localisation. Thus, altered trafficking of an otherwise functional nAChR may underlie the pathogenic effects of these mutations.

## 1. Introduction

Neuronal nicotinic acetylcholine receptors (nAChRs) are a heterogeneous family of ligand-gated ion channels that consist of five transmembrane subunits and are activated by the neurotransmitter acetylcholine (ACh) [1]. Their expression on the surface of neurons and cells is the result of complex and highly regulated processes of subunit synthesis, folding, and assembly in the endoplasmic reticulum (ER), which allow them to reach the Golgi apparatus, followed by the plasma membrane (reviewed in [2]). The essential factors for nAChR assembly were initially discovered by means of forward genetic screening of invertebrates [3], but more recent applications of genetics, proteomics and expression cloning have identified a number of additional molecules and mechanisms that enable the assembly and function of different nAChR subtypes ss (reviewed in [4,5]).

nAChRs assemble into homo- or heteropentamers of α (α2–α7 and α9–α10) and β (β2–β4) subunits [1]; heteromeric receptors have two ACh orthosteric binding sites at the α/β subunit interfaces, whereas homomeric nAChRs, which are composed exclusively of α subunits, have five ACh binding sites [6]. The most prevalent nAChR subtypes in mammalian brains are α4β2 and α7, whereas the main subtype in the peripheral nervous system is α3β4 (reviewed in [1]). However, heteromeric α3β4 nAChRs are also highly expressed in specific brain regions, such as the medial habenula–nucleus interpeduncularis pathway [7], where they are critical modulators of reward-related behaviours [8,9,10]; the olfactory bulb [11]; the hypothalamus [12]; and the inferior colliculus [13], as well as in a variety of non-neuronal cells including cancer cells [14,15].

Because of their roles both in the periphery and in the central nervous system, α3β4 nAChRs have attracted increasing interest as potential drug targets in therapeutic applications for nicotine addiction [8,9,10], respiratory diseases [16], diabetes [17] and cardiac hypertension [18]. Hence, in recent years, a number of groups, including ours, have addressed the pharmacology, as well as the cell biology, of these receptors.

The subunit composition of α3β4 receptors is complicated by their potential to assemble with different subunit stoichiometries, i.e., with two α and three β or with three α and two β subunits; thus, these receptors, as is also the case for the α4β2 subtype, can belong to two different subpopulations depending on the type of subunit in the fifth position. This fifth accessory subunit, although it does not bind ligands, can affect agonist sensitivity, channel kinetics, Ca^2+^ permeability, assembly, and subcellular targeting [19,20].

In our previous studies, we found that chronic nicotine treatment of cultured cells cotransfected with the α3 and β4 subunit cDNAs favours the assembly of receptors with an (α3)_2_(β4)_3_ stoichiometry, resulting in their increased stability and increased export from the endoplasmic reticulum (ER) to the cell surface [21]. In line with the role of the β4 subunit in facilitating transport, we identified an export motif (LFM) in the cytoplasmic loop between the third and fourth transmembrane (TM) domains of the β4 subunit but not in the corresponding loop of the α3 subunit and demonstrated that this motif facilitates export of the assembled pentamer from the ER [21]. In a subsequent study, by transfecting cDNAs coding for β4–α3 dimers together with a cDNA coding for the desired fifth subunit, we were able to generate cells expressing single populations of the receptor with defined stoichiometry [22]. Using this system, we demonstrated that the LFM motif has no role in trafficking when present in the β4–α3 dimer and that it facilitates export only when in the β4 subunit is in the fifth position. We also demonstrated that the β4 subunit in the fifth position has the unique function of regulating the intracellular trafficking of the receptors and their exposure to the surface [22].

A number of missense single-nucleotide polymorphisms (SNPs) in the coding region of the human β4 subunit have recently been identified [23,24]. Interestingly, it has been shown that the overexpression of β4 subunits or the expression of the β4 subunit bearing the T374I variant in the habenula–interpeduncular circuit of mice induces pronounced nicotine aversion and reduces nicotine intake [23,24], whereas the expression of β4 subunits with the R348C variant in the same brain area fails to induce nicotine aversion.

In order to investigate the role of β4 variants, specifically in the fifth accessory β4 subunit, we used the previously developed system to transiently express the β4–α3 dimeric construct together with β4 subunits with or without the D444Y-β4 or R349C-β4 variant (corresponding to the mouse R348C-β4 variant) and studied the intracellular trafficking and cell surface exposure of the resulting pentamers by immunofluorescence and biochemical assays.

## 2. Results

### 2.1. Expression of α3β4 nAChRs Containing a Mutant Accessory β4 Subunit

**Figure 1:**
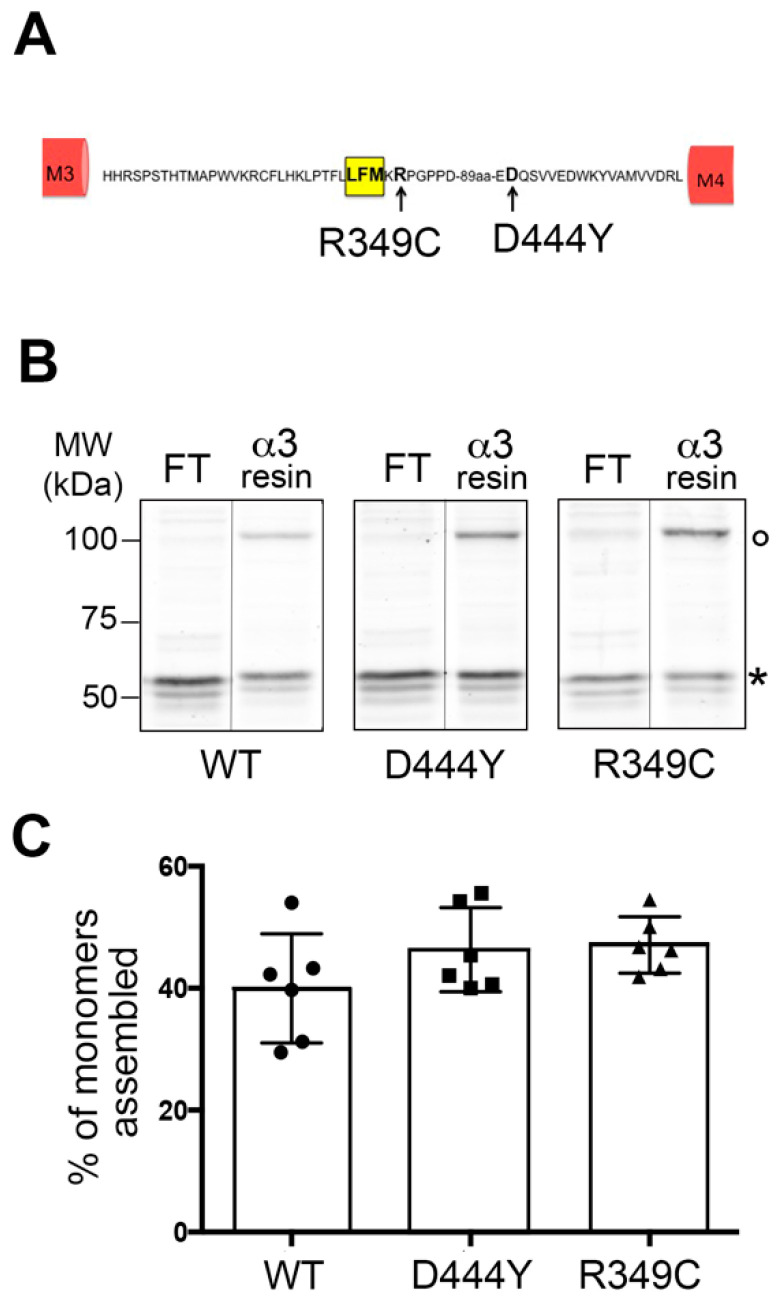
Mammalian cell expression of α3β4 nAChRs with a WT or variant-bearing accessory β4 subunit. (**A**) Schematic representation of the intracellular loop between TM domains 3 and 4 (M3 and M4, respectively; red boxes) of the human β4 subunit. The yellow box indicates the LXM export motif, and the arrows indicate the amino acids modified to obtain the R349C and D444Y variants. (**B**) Assembly of pentamers of α3β4 nAChRs with a WT-β4 or variant-bearing β4 subunit in the fifth position. Lysates of HeLa cells transfected with dimer plus WT-β4 (●), D444Y-β4 (◼) or R349-β4(▲) were passed on a resin covalently linked to anti-α3 antibodies. The bound (α3-resin) and unbound (flowthrough, FT) material was run on SDS-PA gels and stained with anti-β4 antibodies. The circle (○) indicates a band of approximately 110 kDa corresponding to the dimer, and the asterisk (*) indicates a band of approximately 60 kDa corresponding to the β4 monomer. (**C**) Quantification of the percentage of β4 monomers bound to α3-resin assembled in pentamers. In three separate experiments performed in duplicate, there were no statistically significant differences in the assembly of α3β4 nAChRs with a WT-β4, D444Y-β4 or R349C-β4 subunit in the fifth position (one-way ANOVA, Kruskal–Wallis test; three independent experiments with samples in duplicate); MW: molecular weight.

Many of the SNPs identified in the sequence of the human β4 subunit are present in the cytosolic intracellular loop between TM3 and TM4, a portion of the protein that is important in controlling the assembly, trafficking and plasma membrane exposure of nAChRs [25]. In order to test the hypothesis that these mutations affect the assembly and intracellular trafficking of α3β4 nAChRs, we produced two β4 variants bearing the D444Y or R349C mutation by means of site-specific mutagenesis (Figure 1A). As we previously found that the accessory β4 subunit in the fifth position is key for the trafficking of α3β4 receptors with 2α:3β stoichiometry [22], we inserted the mutations only in this subunit. Using our previously described method for obtaining cells expressing α3β4 receptors containing three β4 and two α3 subunits [22], we transfected HeLa cells with a plasmid encoding the β4α3 dimer (DIM) and a plasmid encoding a β4 monomer. The cotransfection of DIM with the WT-β4, D444Y-β4 or R349C-β4 monomer allowed us to obtain cells expressing α3β4 nAChR pentamers with a WT or a mutated β4 subunit in the fifth position.

In three separate experiments performed in duplicate, we first checked whether the presence of mutations in the fifth position of the β4 subunit affects the formation of pentamers. To this end, extracts of cells expressing α3β4 nAChRs were incubated with a resin covalently crosslinked to an anti-α3 antibody. This resin only binds pentamers or unassembled dimers but not the monomeric unassembled β4 subunits that therefore remain in the flowthrough (FT). SDS-PAGE and immunoblotting analyses with anti-β4 antibodies of the FT and α3-resin samples, which contain unassembled and assembled monomers, respectively, were used to measure the efficiency of pentamer assembly (Figure 1B).

Based on the quantification of the β4 subunit signal present in the α3-resin sample, we calculated the percentage of β4 subunits assembled with the DIM to form pentamers. This quantification (Figure 1C and Figure S1) demonstrated that the assembly of all of the pentamers was equally efficient and that the presence of the two β4 mutations did not significantly alter the ability of α3β4 nAChRs to form pentamers.

### 2.2. The Presence of β4 Variants in the Fifth Subunit Alters the Localisation of α3β4 nAChRs

**Figure 2:**
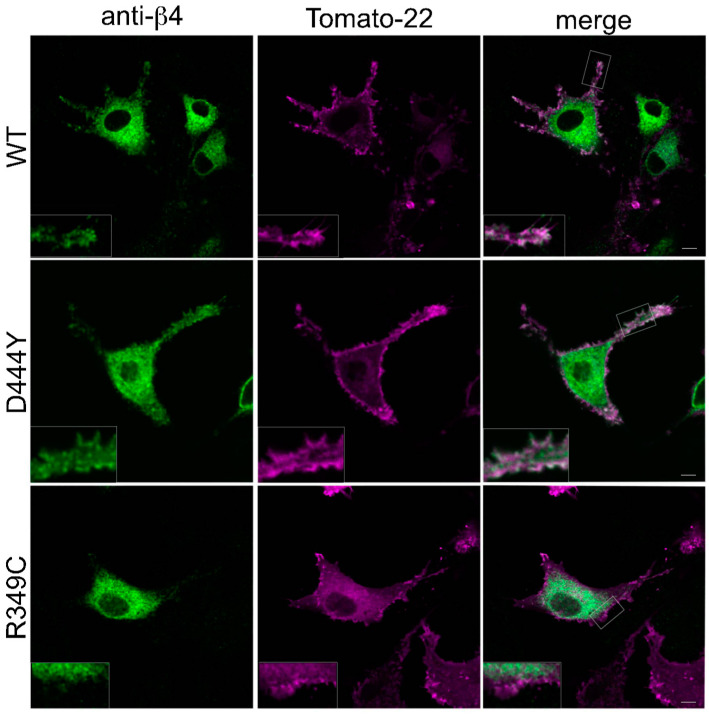
The presence of an accessory R349C-β4 subunit in the pentamers interferes with the surface exposure of nAChRs. NRK cells cotransfected with a plasmid encoding the Tomato-22 protein that marks the plasma membrane plus α3β4 nAChRs with a WT-β4, D444Y-β4 or R349C-β4 subunit in the fifth position. After being incubated at 32 °C for 48 h, the cells were fixed and stained with anti-β4 antibody. The α3β4 nAChRs with a WT-β4 or D444Y-β4 subunit in the fifth position reached the plasma membrane and partially colocalised with Tomato-22, whereas the presence of the R349C-β4 subunit interfered with transport from the ER membrane, and the α3β4 nAChRs were mainly retained in the ER. Representative confocal images with enlarged insets (2.5×). Scale bar: 10 μm.

After verifying that the mutations in the fifth β4 subunit did not affect the assembly efficiency of α3β4 pentamers, we investigated whether they affect the intracellular localisation of α3β4 nAChRs. The nAChR pentamers were expressed in NRK cells, which we had previously identified as being permissive for α3β4 nAChR transport by cotransfecting the β4α3 (DIM) dimer with either WT-β4, D444Y-β4 or R349C-β4 subunits [22], obtaining cells expressing α3β4 nAChRs with WT or mutated β4 subunits in the fifth accessory position. We also cotransfected NRK cells with a plasmid encoding Tomato-22, a fluorescent protein, with a signal for PM localisation [26]. After transfection, the NRK cells were kept at 32 °C (a temperature that favours the intracellular trafficking of nAChRs and their exposure on the cell surface) for 48 h. After fixation, the cells were stained with an anti-β4 subunit in order to reveal the location of the nAChRs. α3β4 nAChRs with WT-β4 or D444Y-β4 in the fifth position reached the cell surface, whereas those with the R349C-β4 subunit in the fifth position were unable to reach the PM and remained in the ER (Figure 2). Note that the R349C mutation is very close to the position of the LFM motif (aa 345-347) that was fundamental for the receptor exit from the ER [24].

**Figure 3:**
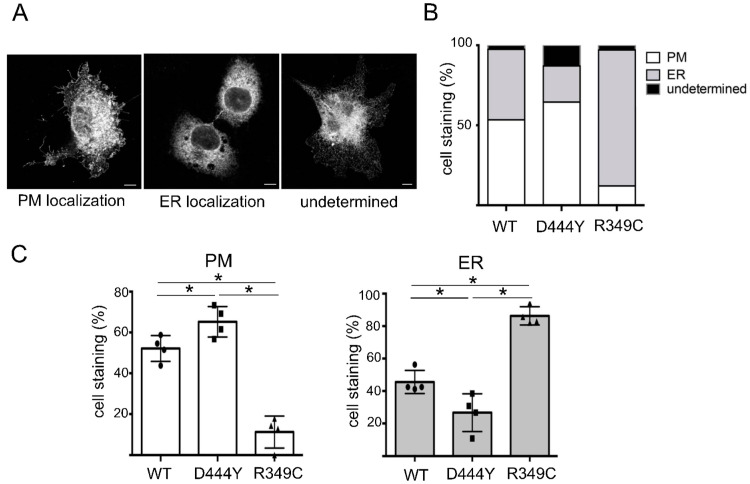
Intracellular localisation of α3β4 nAChRs with a WT or variant-bearing accessory β4 subunit. (**A**) Representative confocal images illustrating anti-β4 plasma membrane (PM), endoplasmic reticulum (ER) or undetermined staining of NRK cells transfected with DIM plus WT-β4, D444Y-β4 or R349C-β4 for 48 h at 32 °C. Scale bar: 10 μm. (**B**) Graph showing the intracellular distribution of nAChRs expressed in NRK cells transfected with DIM plus WT-β4(●)D444Y-β4, (◼) or R349C-β4(▲) for 48 h at 32 °C. Three independent experiments involving 116, 93, and 85 cells, respectively. (**C**) Non-parametric *t* and Mann–Witney tests revealed statistically significant differences in the PM (*) and ER (*) localisations of the three populations with a *p* < 0.05.

In order to quantify the localisation of the three populations of α3β4 nAChR, we examined randomly selected cells expressing different nAChRs and, on the basis of the observed staining, classified them as cells showing a PM signal, cells with exclusive ER staining or cells that could not be easily identified as belonging to either of these categories (undetermined) (Figure 3A). Once again, the presence of the R349C mutation greatly interfered with cell surface exposure; the percentage of cells expressing α3β4 nAChRs in the PM was significantly lower than that of the cells with a WT subunit in the fifth position (Figure 3B,C). Moreover, the distribution of the α3β4 nAChRs with the D444Y-β4 subunit not only confirmed that they could reach the surface but also revealed that the percentage of cells with PM staining was higher than that of cells with a WT subunit, suggesting that this mutation facilitates the arrival of receptors in the PM (Figure 3B,C).

**Figure 4:**
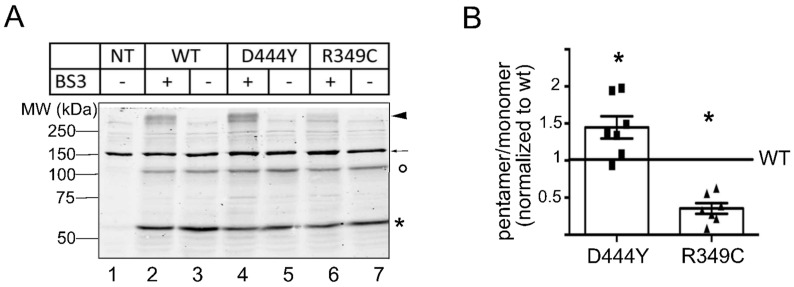
Pentamers containing the D444Y-β4 subunit are enriched in the plasma membrane, but those containing the R349C-β4 subunit are retained intracellularly. (**A**) After 48 h at 32 °C, NRK cells transfected with dimer plus a WT-β4, D444Y-β4 or R349C-β4 subunit were incubated with 2 mM of the impermeable crosslinker BS^3^ for 20 min at 32 °C. After quenching the residual BS^3^ with glycine, the cells were lysed, and sample extracts were run on SDS-PAGE gels. The membranes were stained with anti-β4 antibody (a representative image of the staining is shown). A band corresponding to the molecular weight of the pentamers (≈280 kDa, ◀ arrowhead) and indicating the presence of pentameric α3β4 receptors on the cell surface appeared only in the presence of the crosslinker (lanes 2, 4 and 6). The circle (○) and the asterisk (*) mark the dimer and monomer, respectively; the arrow (←) indicates a non-specific band recognised by the anti-β4 antibody that is also recognised in the lysate of non-transfected NRK cells (NT, lane 1). (**B**) Graph of the quantification of seven independent experiments showing the percentage of pentamers in comparison to the WT. The presence of the D444Y-β4 (◼) subunit significantly increased the number of α3β4 nAChRs on the cell surface (* *p* = 0.031) whereas the presence of the R349C-β4 (▲) significantly decreased the number of α3β4 nAChRs (* *p* = 0.015) (ANOVA, Wilcoxon test).

In order to strengthen our observation that the two mutations have an opposite effect on the intracellular trafficking of α3β4 nAChRs, we quantified the surface-expressed receptors by incubating live cells with the membrane-impermeant crosslinker BS^3^. After quenching with glycine, the cells transfected with dimer plus the WT-β4, D444Y-β4 or R349C-β4 subunit were lysed, and the extracts were analysed by means of SDS-PAGE, followed by immunoblotting. As shown in Figure 4, SDS-PAGE analysis showed that the cells treated with BS^3^ had a high-molecular-weight band (>250 kDa, arrow head, Figure 4A and Figure S2) that corresponded to the molecular weight of the pentamers exposed on the cell surface. This band was only detectable after BS^3^ incubation (see arrow head), and its signal was higher in the samples of cells transfected with DIM + D444Y-β4 than in those transfected with DIM + WT-β4 but very low in those transfected with DIM + R349C-β4, indicating that only the pentamers with WT-β4 or D444Y-β4 in the fifth position efficiently reached the cell surface, whereas those with R349C-β4 were retained intracellularly (Figure 4B).

In line with the imaging data, this biochemical quantification indicates that the presence of the mutations in the β4 accessory subunit in α3β4 nAChRs has a statistically significant opposite effect on the cell surface exposure of the corresponding receptors compared to WT receptors.

## 3. Discussion

Human genetic studies have provided strong evidence for a relationship between genetic variations in the CHRNA5-CHRNA3-CHRNB4 gene cluster and nicotine addiction; moreover, an association study on smoking cessation also suggested that variants in the CHRNB4 gene may decrease craving and withdrawal symptoms [27]. Studies on transgenic mice that overexpressed β4 nAChR subunits in only those neurons that constitutively express β4 subunits showed enhanced nicotine aversion and decreased oral nicotine intake [23]. Similarly, lentivirus-mediated expression in the brains of mice of mutant gain-of-function β4 nAChR subunits, which incorporated CHRNB4 variants associated with reduced risk of tobacco dependence, increased aversion to nicotine [24], whereas transduction with the β4 variant R349C failed to induce nicotine aversion.

Many rare missense variants have been identified in the human β4 subunit, and the role of their expression has been investigated [23,24,28,29]. Among these variants, R349C and D444Y were reported to have an heterozygosity index of 0.014 and 0.005, respectively, and the R349C variant was reported to have a minor allele frequency of 0.0051 [24]. The low allele frequency of an amino acid variant can, by itself, serve as predictor of its functional significance, with a lower frequency indicating greater pathogenicity. R349C is the mutation most frequently encountered in patients affected by sporadic amyotrophic lateral sclerosis [28]. Expression in heterologous systems has shown that when expressed with the WT α3 subunit, the R349C or D444Y variants elicit significantly different nicotine-induced currents from those elicited by WT α3β4 receptors. R349C-β4 subunits significantly reduce nicotine-induced currents [29,30], whereas D444Y-β4 subunits significantly increase nicotine-induced currents [24]. It has also been shown that in comparison with receptors containing WT β4 subunits, those containing the R349C-β4 mutation have similar unitary channel conductance and open time distribution, suggesting that the reduction in nicotine-induced currents is due to a lower density of membrane receptors [29].

All these findings suggest that multiple nicotinic receptor subunit genes likely play a role in the development and maintenance of nicotine dependence and that variants of the β4 gene may have different and, in some cases, opposing functional effects.

In recent years, the integration of human clinical and genetic data with cellular biology data has revealed new forms of diseases not associated with channel biophysical properties but linked to defects in ion channel membrane trafficking and/or post-translational modifications [31]. Therefore, we decided to focus on the trafficking and plasma membrane localisation of receptors containing R349C or D444Y mutations.

We analysed the effect of these mutations in the accessory β4 subunit on the (α3)_2_(β4)_3_ stoichiometry because our previous findings clearly showed that this β4 subunit, which does not participate in the ligand-binding site, has a unique function in regulating the intracellular trafficking of α3β4 receptors, their plasma membrane localisation and their function [22]. In order to obtain the expression of receptors with an (α3)_2_(β4)_3_ stoichiometry, we used our previously developed strategy of coexpressing the β4–α3 dimer with a monomeric β4 subunit [22]. We first checked that the cells expressing α3β4 nAChRs containing the WT or mutated β4 subunit in the fifth position assemble with identical efficiency, indicating that the number of surface receptors does not depend on the number of assembled receptors. We then used immunofluorescence to analyse the intracellular localisation of the pentameric receptors and their plasma membrane expression and found that α3β4 nAChRs with an R349C-β4 subunit were retained intracellularly and that the percentage of cells expressing α3β4 nAChRs on the plasma membrane was significantly lower than that of the cells with a WT subunit in the fifth position. In contrast, α3β4 nAChRs with a D444Y-β4 subunit reached the plasma membrane, and the percentage of cells with plasma membrane staining was higher than that of cells with a WT subunit, suggesting that this mutation facilitates the traffic of the receptors to the PM. These immunofluorescence findings were further confirmed by quantitative crosslinking experiments, which showed that the increased expression of α3-D444Y-β4 receptors on the cell surface was not due to their increased total expression but to increased delivery to the PM.

In the case of the α3β4 subtype, understanding its cell biology is important for diverse human pathologies, including nicotine reward, aversion and dependence [8,9,10]; insulin resistance [17]; and amyotrophic lateral sclerosis [28], in which this subtype is involved. We hypothesise that the presence of a rare variant in one allele could be sufficient to alter, by a dominant negative mechanism, the localisation and consequent function of this receptor subtype, which plays such important roles in the physiology of the peripheral and central nervous systems.

Our results demonstrate that the presence of R349C and D444Y mutations in the fifth position of the human β4 subunit deeply alter the α3β4 receptor functionality by altering its localisation; the mechanism involved in this alteration is under study. It has previously been demonstrated that the ALS-linked R349C mutation in the β4 subunit of the α4β4 receptor subtype [28,29] is very close to the LFM motif and decreases the rate of fusion between α4β4 receptor-containing vesicles and the plasma membrane (with only a small decrease in the number of receptors per vesicle), resulting in a lower frequency of agonist-induced currents. It also diminishes the number of ER exit sites, suggesting that the reduced receptor insertion into the plasma membrane is due to a change in the early stage of trafficking [30]. As the LXM motif in the β4 subunit is a key to the outwardly directed trafficking through the secretory pathway and adjacent to the R349C-β4 mutation, it is possible that a nearby disruption in the amino acid sequence could affect LFM function, interfering with its interaction with Sec24D and the consequent recruitment of the receptor at the level of ER exit sites.

In the case of the D444Y-β4 mutation, a charged aspartic acid is substituted by a non-polar tyrosine that can be phosphorylated by a number of kinases. The phosphorylation of nAChRs can have a wide variety of effects, ranging from alterations in surface expression to synaptic targeting and receptor desensitisation [32,33]. It is not known whether the change in amino acids in α3-D444Yβ4 receptors affects the conformation of the subunit/receptor or whether the presence of tyrosine and its phosphorylation allows for the binding of a chaperone protein that increases the surface expression of the receptor.

In conclusion, our findings show that the presence of missense variants exclusively in the fifth subunit of the α3β4 subtype alters its trafficking and plasma membrane localisation, further confirming the major role of this accessory subunit in the trafficking of the α3β4 subtype previously reported by our group [22].

## 4. Materials and Methods

### 4.1. Plasmid Constructions

The dimeric construct (DIM) plasmid coding for a human β4 subunit followed by a human α3 subunit was preiously described in [22]. The plasmid encoding tomato-22, which was used as the plasma membrane (PM), marker was described by Fossati et al. [26]. The mutants D444Y and R349C were generated by site-directed mutagenesis using a Quikchange Lightining site-directed mutagenesis kit (#210519, Agilent). The template used was pCDNA3-h β4 (a kind gift of Sergio Fucile, “Sapienza" University of Rome), which codes for the entire coding sequencing (CDS) of the human β4 subunit (protein sequence, ACHB4_HUMAN, UniProt database). To obtain the D444Y construct, we substituted D at position 444 with Y using the following oligonucleotides (the mutated nucleotide is indicated in bold):

forward: 5′-GAAGAATGACGATGAA**T**ACCAGAGTGTCGTTGAGG-3′

reverse: 5′-CCTCAACGACACTCTGGT**A**TTCATCGTCATTCTTC-3′

To obtain the R349C construct, we substituted R at position 349 with C using the following oligonucleotides (the mutated nucleotide is indicated in bold):

forward: 5′-CCTTCCTCTTCATGAAG**T**GCCCTGGCCCCGACAGC-3′

reverse: 5′-GCTGTCGGGGCCAGGGC**A**CTTCATGAAGAGGAAGG-3′

### 4.2. Antibodies

The α3 and β4 pAbs were previously described in [7]. Rabbit Alexa Fluor 488 and 543 were acquired from Invitrogen (Waltham, MA, USA), and mouse IgG DyLight 549 was acquired from Pierce/Thermo Fisher (Waltham, MA, USA). Infrared-conjugated IgG IRDye 800CW and IgG IRDye 680CW were acquired from LI-COR Bioscience (Lincoln, NE, USA).

### 4.3. Cell Culture and Transfection

Normal rat kidney epithelial (NRK) cells were purchased from ATCC-LGC Standards (London, UK). HeLa and NRK cells were maintained in DMEM (Dulbecco’s Modified Eagle Medium) supplemented with 10% foetal bovine serum, 1% l-glutamine and 1% penicillin/streptomycin at 37 °C in a 5% CO_2_ humidified atmosphere. HeLa cells were transiently transfected with the use of JetPEI (Polyplus-transfection SA, Illkirch-Graffenstaden, France) as previously reported in [22]. Transient transfection of NRK cells was performed with Lipofectamine 3000 (Thermo Fisher Scientific, Waltham, MA, USA). Equimolar amounts of plasmids coding for dimeric and monomeric constructs were used. A total of 180,000 cells plated in a 12-well plates were transfected using 3 μg of cDNA and 9 μL of Lipofectamine (DNA/transfecting reagent ratio, 1:3) per well. After 24–48 h at 32 °C, cells were lysed, fixed or incubated with the crosslinker BS^3^ bis(sulfosuccinimidyl)suberate (Thermo Fisher Scientific, Waltham, MA, USA).

### 4.4. Biochemical Analysess

Sodium dodecyl sulphate–polyacrylamide gel electrophoresis (SDS-PAGE) and immunoblotting were carried out according to standard procedures. Primary antibodies were revealed by infrared-conjugated IgG IRDye 800CW or IgG IRDye 680CW (LI-COR Bioscience, Lincoln, NE, USA). Blots were scanned with an Odyssey CLx infrared imaging system (LI-COR Biosciences, Lincoln, NE, USA), and band intensities were determined with Image Studio software, version 2.1.10 (LI-COR Biosciences, Lincoln, NE, USA).

### 4.5. Immunofluorescence and Image Analysis

Cells grown on coverslips were fixed with 4% paraformaldehyde, permeabilised with Triton X-100 and processed for immunofluorescence. Confocal images were taken with an LSM 800 ZEISS confocal microscope using a 63× PlanApo lens.

For quantification of the percentage of cells with β4 PM staining, random fields of transfected cells were acquired with a 40× PlanApo lens using an LSM 800 ZEISS confocal microscope (Carl ZEISS, Oberkochen, Germany). The cells were then distributed in three categories: cells that clearly showed plasma membrane staining, cells that exhibiting endoplasmic reticulum staining and cells that could not be easily identified as belonging to either of these two categories (undetermined). Based on the number of cells assigned to each category, we evaluated the ER and PM localisation of receptors having in the fifth position WT-β4 or D444Y-β4 or R349C-β4 subunits.

### 4.6. Immunoprecipitation Using Cross-Linked Resins

Affinity-purified anti-α3 antibodies were immobilised to packed Protein A-Sepharose beads (GE Healthcare, Chicago, IL, USA) by dimethyl-pimelimidate-mediated crosslinking [34]. HeLa cells were transfected with the dimeric construct (DIM) plus the accessory subunit; 24 h thereafter, cells were lysed with a lysis buffer (PBS, 2% Triton-X100 with protease inhibitors) for 30 min at 4 °C. PNSs (post-nuclear supernatants) were recovered after centrifugation at 2000× *g* for 10 min at 4 °C and incubated O/N at 4 °C with the immobilised antibodies. The flowthrough from the resin (FT) containing unassembled monomers and the material bound to the resin (α3-resin) containing the unassembled DIM plus assembled pentamers were run on SDS-PA gels. To estimate the efficiency of pentamer assembly, we calculated the percentage of monomers incorporated into pentamers from the ratio of the monomers in the α3-resin lane to the total amount of monomers (the sum of monomers in the α3-resin and FT lanes).

### 4.7. Crosslinking Analysis

Crosslinking on transfected cells was performed as described in [35]. The concentration of BS^3^ used was 2 mM (from a freshly prepared stock solution in 5 mM Na citrate, pH 5.5). Samples were loaded on 7% SDS-PA gels and blotted in the presence of 0.037% SDS.

## Data Availability

Details regarding methods, results and data analysis can be obtained by contacting Sara Francesca Colombo.

## Supplementary Materials

The following supporting information can be downloaded at: https://www.mdpi.com/article/10.3390/molecules28031247/s1, Figure S1. Assembly of pentamers of α3β4 nAChRs with a WT-β4 or variant-bearing β4 subunit in the fifth position. Figure S2. Pentamers containing the D444Y-β4 subunit are enriched at the plasma membrane, but those containing the R349C-β4 subunit are retained intracellularly.

## Sample Availability

Samples of the compounds, plasmids coding for DIM, β4 WT, D444Y-β4 or R349C-β4 are available from the authors.
