# Supplementary material for: Rare Missense Variants of the Human β4 Subunit Alter Nicotinic α3β4 Receptor Plasma Membrane Localisation
Source: Molecules. 2023 Jan 27;28(3):1247. doi: 10.3390/molecules28031247 (PMC9919425; doi:10.3390/molecules28031247)
Supplement: Supplementary file 1 [file molecules-28-01247-s001.zip › molecules-2171791-supplementary.pdf]

Supplementary Material

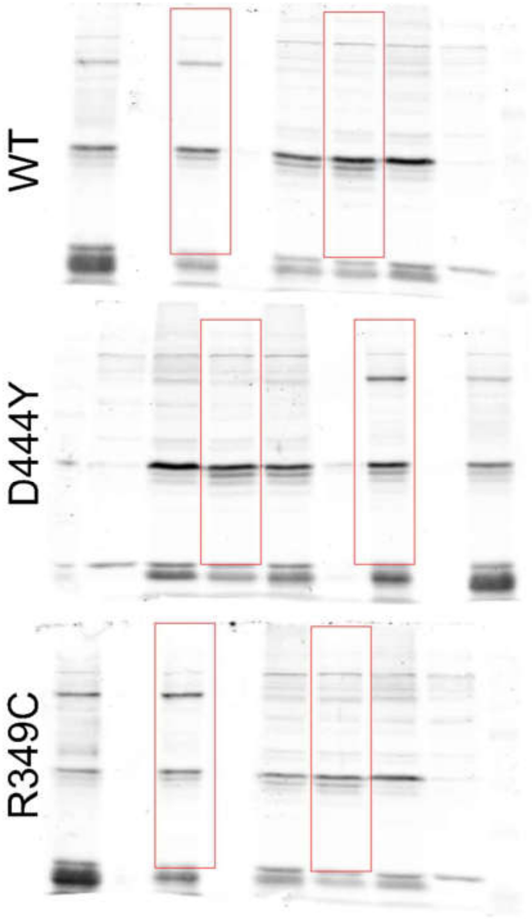

Figure S1. Uncropped figure 1B Assembly of pentamers of $\alpha3\beta4$ nAChRs with a WT-β4 or-variant -bearing β4 subunit in the fifth position. Lysates of HeLa cells transfected with dimer plus WT-β4, D444Y-β4 or R349-β4 were passed on a resin covalently linked to anti-$\alpha3$ antibodies. The bound ($\alpha3$-resin) and unbound (flow-through, FT) material was run on SDS-PA gels and stained with anti-β4 antibodies.

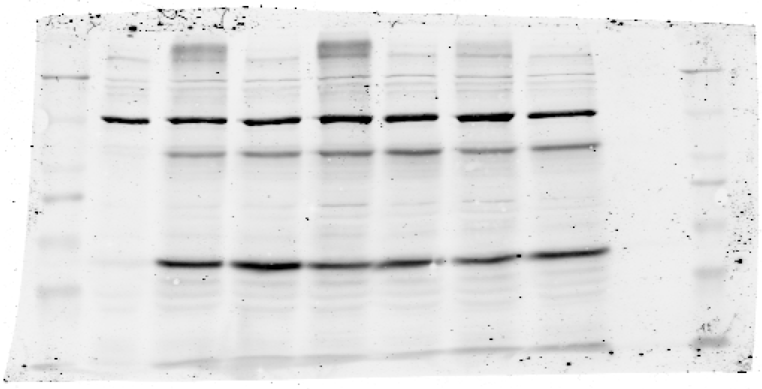

Figure S2. Uncropped figure 4A. Pentamers containing the D444Y-β4 subunit are enriched at the plasma membrane, but those containing the R349C-β4 subunit are retained intracellularly. After 48 h at 32 °C, NRK cells transfected with dimer plus a WT-β4, D444Y-β4, or R349C-β4 subunit were incubated with 2 mM of the impermeable cross-linker BS^3 for 20 min at 32 °C. After quenching the residual BS^3 with glycine, the cells were lysed and sample extracts were run on SDS-PAGE gels. The membranes were stained with anti-β4 antibody (a representative image of the staining is shown).
